# P-2018. A Digital Leap in Infection Surveillance: Evaluation of a Mobile App for Real-Time Reporting of HAIs and Near Misses in a Tertiary Care Hospital

**DOI:** 10.1093/ofid/ofaf695.2182

**Published:** 2026-01-11

**Authors:** Maria Tom, Jomel Raju, Leena James

**Affiliations:** St. Joseph's College of Pharmacy, Cherthala, Kochi, Kerala, India; St. Joseph's College of Pharmacy, Cherthala, Kochi, Kerala, India; St. Joseph's College of Pharmacy, Elampally, Kerala, India

## Abstract

**Background:**

Delayed and underreported healthcare-associated infections (HAIs) and near misses undermine infection control in resource-limited hospitals. This study evaluated the impact of a real-time mobile reporting application on the volume, speed, and quality of HAI surveillance in a tertiary care hospital in South India.

**Figure:**
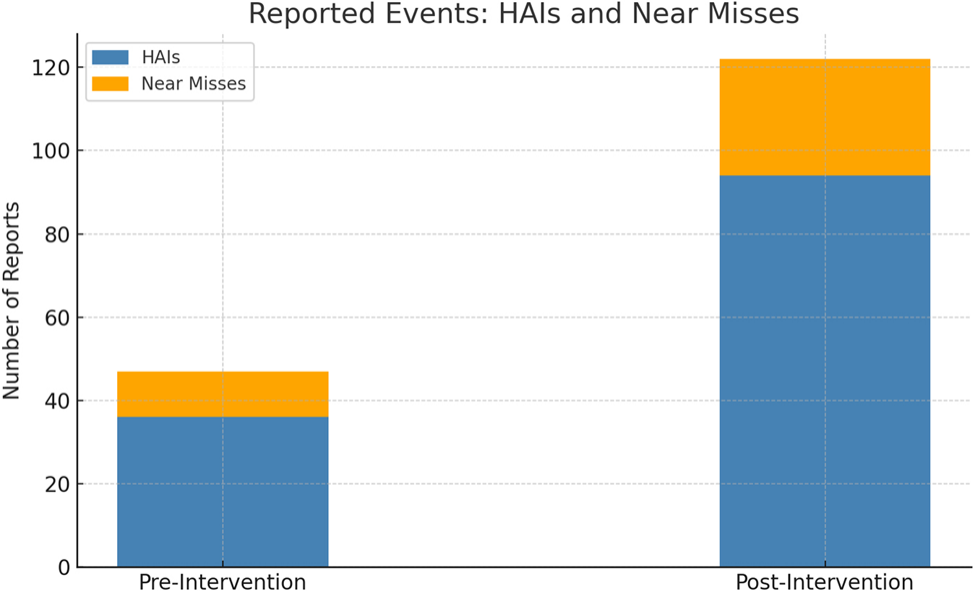
Reported Events: HAIs and Near Misses Before and After Mobile App Implementation This stacked bar chart illustrates the increase in reported healthcare-associated infections (HAIs) and near miss events during the 6-month period following the introduction of a mobile-based reporting application in a tertiary care hospital. The number of HAI reports increased from 36 to 94, and near miss reports rose from 11 to 28. The digital platform enhanced frontline engagement and reporting frequency.

**Figure:**
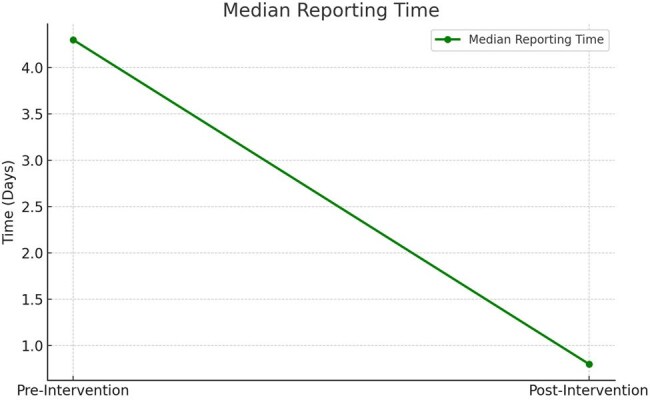
Reduction in Median Reporting Time Following Digital Surveillance Implementation This line chart shows the substantial improvement in reporting timeliness for HAIs and near misses. Median time from event occurrence to submission decreased from 4.3 days to 0.8 days post-intervention. The mobile app’s real-time functionality, QR login, and simplified interface contributed to this rapid acceleration in report turnaround time.

**Methods:**

A prospective, mixed-methods pilot study was conducted from January to June 2024 in a 650-bed tertiary teaching hospital. A multilingual mobile app featuring dropdowns, incident categories (CLABSI, CAUTI, VAP, SSI), image upload, and offline sync was deployed in five high-risk departments. A total of 180 nurses and 60 doctors received structured training. Pre-post comparisons used chi-square and Wilcoxon signed-rank tests. User feedback was measured using the System Usability Scale (SUS) and Net Promoter Score (NPS).

**Figure:**
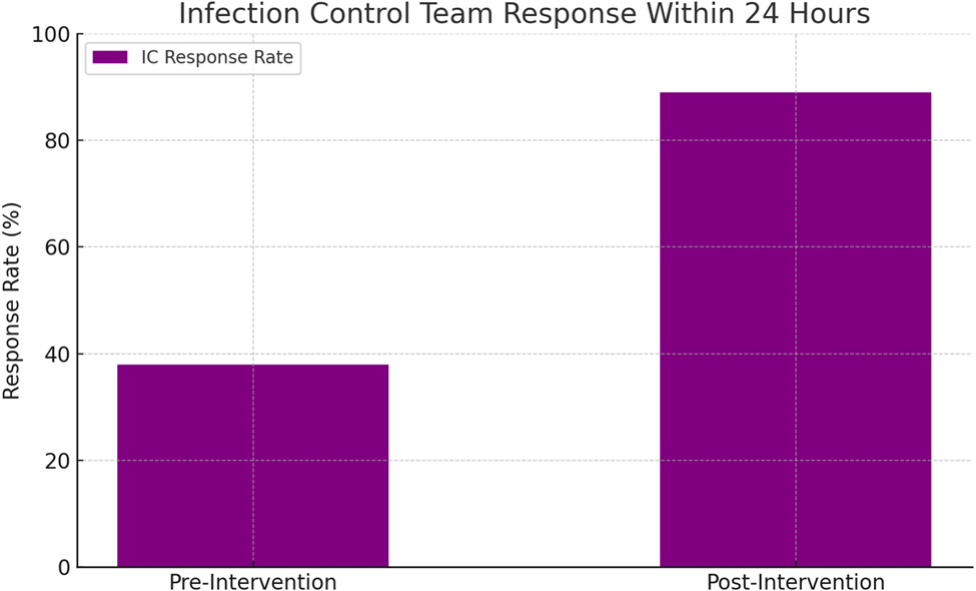
Infection Control Team Response Within 24 Hours: Pre vs. Post Intervention This bar chart compares the infection control team’s response rate within 24 hours before and after the mobile app implementation. Timely response increased from 38% to 89% post-intervention (p < 0.001), reflecting enhanced alert visibility, automated notifications, and accountability triggered by real-time data capture.

**Results:**

Over 6 months, the app facilitated 94 HAI and 28 near miss reports, a 2.6× increase in reporting frequency compared to the previous 6-month period (p < 0.001). Median time to report decreased from 4.3 days to 0.8 days (p < 0.001). The app achieved a mean SUS score of 84.5 and NPS of +61, indicating excellent usability and strong user acceptance. Timely infection control team response within 24 hours improved from 38% to 89% (p < 0.001). Users reported improved accountability, efficiency, and ease of reporting.

**Conclusion:**

The mobile app significantly improved the speed, quantity, and quality of HAI and near miss reporting in a resource-constrained Indian hospital. This model demonstrates the feasibility and scalability of digital tools in strengthening infection surveillance and patient safety practices in low-resource healthcare systems.

**Disclosures:**

All Authors: No reported disclosures

